# Individualized Hemodynamic Management in Newborns

**DOI:** 10.3389/fped.2020.580470

**Published:** 2020-10-07

**Authors:** Willem P. de Boode

**Affiliations:** Division of Neonatology, Department of Pediatrics, Radboud University Medical Center, Radboud Institute for Health Sciences, Amalia Children's Hospital, Nijmegen, Netherlands

**Keywords:** hemodynamics, individualized medicine, neonatology, shock, hypotension, patent ductus arteriosis, persistent pulmonary hypertension in the newborn (PPHN)

## Abstract

In our aim to improve patient outcome we are transitioning from a “one-size-fits-all” protocolized approach toward an individualized hemodynamic management, that is tailored to the cardiovascular (patho-)physiology and the specific clinical characteristics of each individual patient. In this narrative review an overview is provided about an individualized approach toward various neonatal hemodynamic conditions.

## Introduction

Newborns admitted to the neonatal intensive care unit have an increased risk of hemodynamic failure for several reasons. The immaturity of the cardiovascular system in preterm infants, combined with impaired compensatory mechanisms, makes them prone to injury related to hypoperfusion and hypoxemia. Many conditions may affect the heart and vasculature, for example perinatal hypoxia-ischemia, sepsis, necrotizing enterocolitis, air leakage syndromes, and congenital anomalies, such as structural heart defects and congenital diaphragmatic hernia. Moreover, well-intended therapeutic interventions could potentially have adverse hemodynamic effects, for instance invasive ventilation with high mean airway pressure impeding venous return, iatrogenic tachycardia secondary to chronotropic properties of cardiovascular drugs limiting the filling phase (preload) of the heart, and drug-induced systemic vasodilation in case of sedation, analgesia, and muscle relaxation. It is therefor imperative that cardiovascular disturbances are detected as soon as possible or preferably prevented. The fact is, however, that this is easier said than done. It has been shown that severe systemic hypoperfusion can remain clinically undetected, irrespective of the level of experience of the health care professional (1, 2). This is probably related to the fact that hemodynamic monitoring and management is generally based on the interpretation of indirect markers of systemic perfusion, such as heart rate, blood pressure, urine output, blood gas analysis, capillary refill time, “peripheral perfusion” et cetera (3). For decades hemodynamic management could be classified as a “pressure-based approach,” in which the presence of hypoperfusion was falsely predominantly evaluated by monitoring of blood pressure. This has resulted in a “one-size-fits-all” protocol with hypotension as main criterium to intervene, that has been used by many (4).

**Figure 1:**
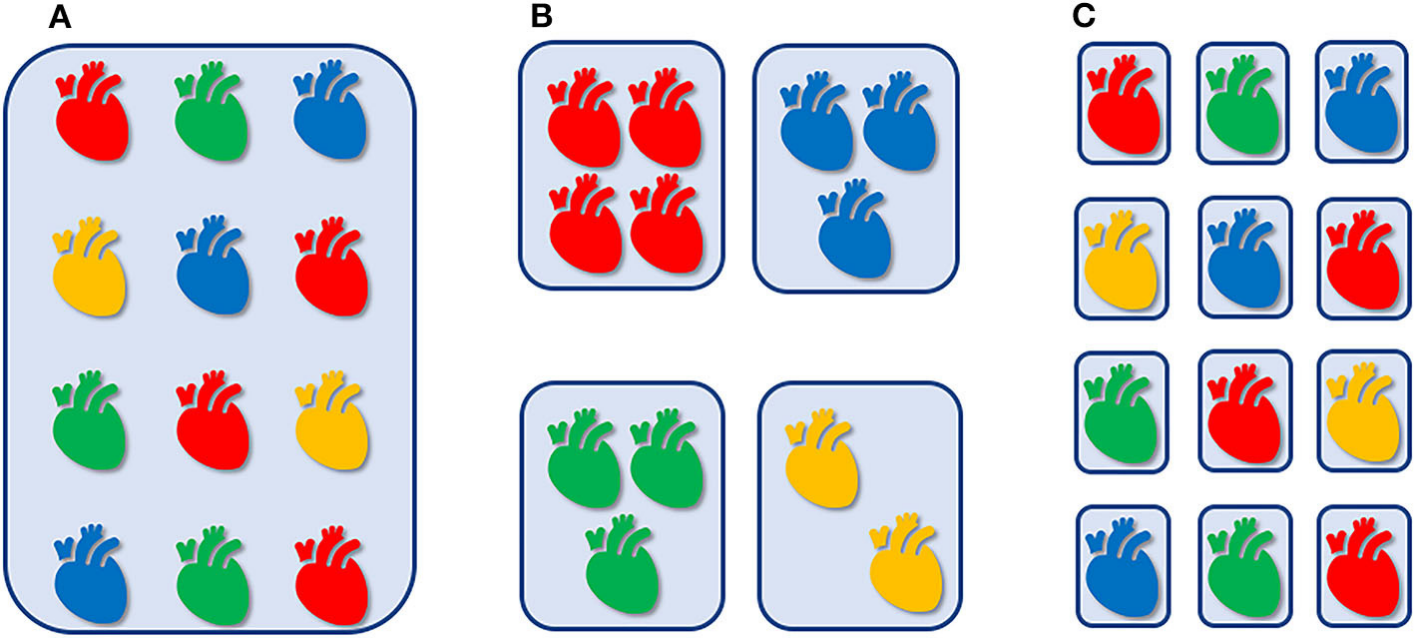
Empirical, “one-size-fits-all” approach, irrespective of cardiovascular physiology and clinical context **(A)**. Hemodynamic management that is stratified by presumed underlying pathophysiology **(B)**. Individualized hemodynamic management that is tailored to the cardiovascular (patho-)physiology and the specific clinical characteristics of each individual patient **(C)**.

Nowadays, we are transitioning from an empirical approach via stratified medicine toward an individualized hemodynamic management (Figure 1). Not all newborn infants with hemodynamic instability are treated in a similar (“protocolized”) manner (Figure 1A), since we stratify patients based upon presumed underlying pathophysiological mechanisms (diagnosis) and adapt our therapeutic interventions accordingly (Figure 1B). For example, systemic hypotension is treated different in an extreme preterm infant in transition, than in a full-term newborn with perinatal hypoxic-ischemic encephalopathy or congenital diaphragmatic hernia, or in a patient after surgery for severe necrotizing enterocolitis. However, not all patients within the same diagnostic category necessarily share similar etiologic factors related to alike hemodynamic disturbances. As an example, systemic hypotension in a septic patient could be caused by either myocardial impairment or systemic vasodilation secondary to cardiodepressive or vasoplegic action of cytokines, respectively, requiring a totally different cardiovascular intervention. In other words, an individualized hemodynamic management is indicated, that is tailored to the cardiovascular (patho-)physiology and the specific clinical characteristics of each individual patient (Figure 1C).

Several aspects of personalized hemodynamic management will be discussed for patients with highly prevalent hemodynamic conditions, namely persistent patent ductus arteriosus (PDA), shock/hypotension, and persistent pulmonary hypertension of the newborn (PPHN).

## Patent Ductus Arteriosus

One of the most controversial issues in neonatology is whether or not a persistent PDA should be treated. Although a PDA is associated with serious adverse outcomes, such as mortality, pulmonary hemorrhage, bronchopulmonary dysplasia, intraventricular hemorrhage, and necrotizing enterocolitis, firm evidence is currently still lacking for a causal relationship, despite many clinical trials. The question remains whether a PDA should be considered a binary entity in (extreme) preterm infants, that should either be treated and actively closed or can be managed conservatively awaiting spontaneous closure^.^(5). Given the high spontaneous closure rate in preterm infants born after more than 28 weeks' gestation, there is generally no routine screening of the PDA performed in this subpopulation. From a pathophysiological perspective it would be highly informative to objectively quantify the transductal left-to-right shunt volume, since this would enable triage of preterm infants and identify those that could benefit from active medical closure of the ductus arteriosus. Higher transductal shunt volumes are associated with increased pulmonary and decreased systemic perfusion, that possibly explains associated morbidities as pulmonary hemorrhage, high need for respiratory support, bronchopulmonary dysplasia on the one hand and necrotizing enterocolitis, cerebral injury on the other hand. Neonatologist Performed Echocardiography (NPE) is used to estimate the degree of transductal shunt volume and determine the hemodynamic significance of the PDA (6, 7). Several scoring systems, incorporating clinical, and/or echocardiographic characteristics, have been published to stage the severity (hemodynamic significance) of the PDA (8–10). These scoring systems are used to estimate the risk of mortality or chronic lung disease in preterm infants with a PDA with a positive and negative predictive value of 92 and 82%, respectively (9).

When it is decided to treat a PDA in a preterm infant, NPE can be used to monitor the effect of the therapeutic intervention. It has been shown that the intensity of treatment with cyclooxygenase-inhibitors can safely be reduced by evaluating the vasoconstrictive action on the ductus arteriosus. Su et al. performed a non-blinded randomized controlled trial of conventional vs. echocardiography-guided treatment of a PDA with indomethacin in 93 preterm infants <1,500 g (11). In the echocardiography-guided study arm, an additional dose of indomethacin was only given if the transductal flow pattern persisted to be classified as “pulsatile” or “growing,” suggestive of high transductal shunt volume. This resulted in a significant reduction in number of doses indomethacin (3.2 ± 1.4 vs. 1.6 ± 0.9 doses; *p* < 0.01) and less side-effects without causing a difference in ductal closure rate or obvious effects on mortality or incidence of chronic lung disease. In two similar studies, a subsequent dose of cyclooxygenase-inhibitor was only prescribed when ductal constriction was considered insufficient by evaluating the change in transductal diameter (12, 13). In the first, a next dose of indomethacin was only administered if the transductal diameter was more than 1.6 mm. This approach reduced the total number of doses indomethacin from a median of three to only one without any increase in the incidence of ductal closure failure, ductal reopening, surgical ligation, pulmonary hemorrhage, chronic lung disease, necrotizing enterocolitis, or renal failure (12). Bravo et al. showed in a randomized trial that echocardiography-guided treatment with ibuprofen, i.e., only administration of a next dose of ibuprofen if the transductal diameter measured more than 1.5 mm, reduced the number of doses from a median of three [IQR 3–4] to two [IQR 1–5.7] (13). No differences in ductal closure failure, mortality of major neonatal morbidity was observed.

In addition to classification of the severity of transductal shunt volume and optimization of dosing schemes of indomethacin or ibuprofen, NPE is helpful in anticipating and identifying of complications of surgical ligation of the ductus arteriosus, such as post ligation cardiac syndrome (PLCS). PLCS is characterized by systemic hypotension secondary to myocardial impairment, oxygenation failure, and increased need of ventilatory support. Jain et al. observed that a left ventricular output <200 mL/kg/min in patients 1 h after ductal ligation was predictive of low cardiac output 8 h post-surgery (sensitivity (Se) 1.0; specificity (Sp) 0.89), systemic hypotension (Se 0.83; Sp 0.72), and need for inotropes (Se 1.0; Sp 0.65) (14). In a subsequent epoch study echocardiography was used to anticipate on PLCS and if a LVO <200 mL/kg/min was measured 1 h after ligation, milrinone was started in an attempt to prevent a low cardiac output state (14). This echocardiography-guided management resulted in a lower incidence of ventilatory failure (15 vs. 48%; *p* < 0.05), less inotropic support (19 vs. 56%; *p* < 0.05), and a trend toward less oxygenation failure (26 vs. 52%; *p* = 0.08). Despite the prophylactic use of milrinone, it was observed that about 50% of patients still developed PLCS, reason why other echocardiographic parameters are studied as potential preemptive indicators of myocardial impairment. For example, a prolonged isovolumic relaxation time (IVRT) in the preoperative phase, indicating myocardial diastolic dysfunction, is associated with an increased risk of PLCS (15).

As illustrated, hemodynamic management of preterm infants with a PDA can be adapted according the specific cardiovascular physiology and clinical context of the individual patient. In this way it is possible (1) to predict the hemodynamic significance of the PDA, that is associated with an increased risk of chronic lung disease and mortality, (2) to potentially reduce the number of doses of cyclooxygenase-inhibitors, and (3) to anticipate and prevent complications after surgical ligation of the ductus arteriosus.

## Shock/Hypotension

The terms shock and hypotension are obviously not interchangeable. Shock is defined as a condition characterized by an imbalance between oxygen demand and delivery on a cellular level. Oxygen delivery is mainly determined by hemoglobin concentration, arterial oxygen saturation, and cardiac output. When the amount of oxygen delivered to the peripheral tissues doesn't fulfill the demands, compensatory mechanisms will aim to redistribute blood to the vital organs in an attempt to prevent these from hypoxemic-ischemic injury. Three stages of shock are distinguished, namely (1) compensated shock, (2) uncompensated shock, and (3) irreversible shock. In the first stage, neuroendocrine compensatory mechanisms are responsible for preservation of blood (and oxygen) supply to the brain, the heart and the adrenal glands. This phase is called the compensated shock and is characterized by selective constriction of the vasculature perfusing the non-vital organs, such as kidneys, intestines, muscles, liver and skin. By the counteracting effects of constriction of non-vital organ vasculature and dilation of vessels supplying the vital organs, blood pressure can remain unaffected. There is no linear relation between cardiac output and blood pressure, as can be understood from Hagen-Poiseuille's Law. According this law, cardiac output is determined by the pressure gradient (arterial blood pressure minus right atrial pressure) divided by the systemic vascular resistance. This means that blood pressure can be in the normal (or even high) range during a state of low cardiac output provided that vascular resistance is increased. Systemic hypotension will occur when compensatory vasoregulatory mechanisms fail, known as the uncompensated phase of shock. Relying solely on the level of blood pressure, a “pressure-based” approach, might lead to misinterpretation of the hemodynamic status of newborns and the initial, compensated phase of shock might be missed. Neglecting the possibility of the presence of a compensated shock might have detrimental effects. There are indications that in fetuses and very preterm infants the blood vessels perfusing the cortex are not acting as high-priority vasculature and therefore not dilating in response to decreased blood flow, as would be expected for a vital organ as the brain (16–18). One might speculate that this observation could be (partially) responsible for the fact that survival in (extreme) preterm infants is increasing without an equivalent reduction in neurodevelopmental impairment (19–23).

Hence, it is imperative to detect low cardiac output already in the compensated stage of shock and not wait for intervention after systemic hypotension occurs, suggestive for the uncompensated stage of shock. This requires comprehensive hemodynamic monitoring with at least simultaneous measurement of arterial blood pressure and cardiac output. Under normal physiological circumstances both systemic blood flow and blood pressure are in the normal reference range. The combination of low cardiac output with a normal or high blood pressure suggest a compensated shock, whereas in a uncompensated shock both blood pressure and cardiac output are in the lower ranges (24). Measurement of cardiac output is feasible and applicable in (preterm) newborn infants, such as transthoracic echocardiography, electrical biosensing technologies (bioimpedance and bioreactance), and transpulmonary ultrasound dilution (25). Interpretation of both cardiac output and arterial blood pressure enables the selection of the optimal cardiovascular drug that is based on the presumed etiology of impaired perfusion and oxygenation. For this it is imperative that the clinician is aware of the pharmacokinetic and pharmacodynamic characteristics of the available drugs. Algorithms are available for this purpose (24). Comprehensive hemodynamic monitoring has been shown to potentially improve outcome. Integrating clinical assessment with NPE and regional (cerebral and intestinal) oxygenation monitoring using near infrared spectroscopy resulted in a shorter time to clinical recovery in critically ill infants with cardiovascular compromise (26, 27).

## Persistent Pulmonary Hypertension of the Newborn

Increased pulmonary blood pressure can be the consequence of either an increase in pulmonary blood flow (e.g., congenital heart disease with large left-to-right shunting), pulmonary wedge pressure (left ventricular failure; pulmonary vein stenosis), or pulmonary vascular resistance. Increased pulmonary vascular resistance is caused by maladaptation (reactive vasoconstriction on various stimuli), maldevelopment (remodeling of blood vessels), or underdevelopment (hypoplasia) of the pulmonary vasculature. The typical clinical presentation of a patient with PPHN is hypoxemic respiratory failure with sometimes signs of transductal right-to-left shunting (difference in pre- and postductal oxygen saturation ≥5%) and systemic hypotension. PPHN is associated with both left and right ventricular failure (28–32). Increased right ventricular (RV) afterload potentially leads to impaired systolic and diastolic myocardial impairment. Left ventricular (LV) preload is impeded by dilation of the right ventricle, deceased right ventricular output and right-to-left transductal shunting, resulting in decreased left ventricular output. Hypoxia and acidosis may further deteriorate myocardial performance. It is important to be informed about (imminent) ventricular failure in patients with PPHN, since treating systemic hypotension by increasing systemic vascular resistance might further impair myocardial performance, resulting in low cardiac output. Sehgal et al. found biventricular failure (ventricular output <150 mL/kg/min) in 67% of patients with PPHN (RV and LV failure in 64 and 76%, respectively) (29). In 70% of these patients signs of RV diastolic dysfunction was observed. NPE can be used to assess the severity of PPHN and estimate the risk of mortality or need for support with extracorporeal membrane oxygenation (ECMO) in order to timely transfer the patient to an ECMO center. Aggarwal studied the predictive value of the S/D-ratio, an indicator of systolic and diastolic global RV function reflecting ventricular loading and contractility (30). This ratio is calculated from the Doppler signal of tricuspid regurgitation, dividing the systolic duration by the diastolic duration within one heart cycle. An RV S/D>1.3 is associated with mortality or need for ECMO in patients with PPHN with a positive and negative predictive value of 68 and 81%, respectively. Advanced echocardiographic modalities can also stratify PPHN patients and predict adverse outcomes. In a retrospective study in 86 patients born after ≥ 35 weeks' gestation, diminished tricuspid annular plane systolic excursion (TAPSE) <4 mm and global longitudinal peak strain (GLPS) ≥9% was associated with the need for ECMO of risk of death.

In a case report pulmonary vasodilation could successfully be evaluated in a patient with pulmonary hypertension with RV failure with the use of speckle tracking echocardiography (33). In a retrospective observational study in 20 preterm infants with prolonged preterm rupture of membranes (PPROM) before 24 weeks' gestation, Shah and Kluckow showed that NPE identified the presence of PPHN earlier and enabled targeted treatment with nitric oxide inhalation (iNO) (34). This approach resulted in improved survival in this severely affected population. It is known that not all patient with congenital diaphragmatic hernia (CDH) respond successfully to iNO and its use is discouraged, based on the results of the NINOS trial, in which an increased mortality and ECMO requirement was observed in the iNO-treated patients (35). However, a recent study showed that the non-response to iNO in patients with CDH might be related to the presence of LV failure (36). Hypoplasia or myocardial dysfunction of the LV is more often observed in newborn infants with CDH. Decreasing pulmonary vascular resistance with iNO will augment pulmonary venous return, that might further deteriorate LV function. It was concluded that iNO treatment improved oxygenation and reduced the need for support with ECMO in CDH patients with preserved LV systolic function (36).

Being informed about myocardial performance is important in patients with PPHN, since hemodynamic management will be quite different in the presence of ventricular failure.

## Individualized Hemodynamic Management

The clinical scenarios discussed above illustrate the importance of a comprehensive hemodynamic assessment in order to timely diagnose cardiovascular failure, elucidate underlying pathophysiology, guide hemodynamic therapy, monitor the effects of the interventions, and adjust treatment as appropriate. This enables an individualized hemodynamic management that is optimized for the patient's specific pathophysiology and clinical situation. However, this personalized hemodynamic approach is more complex than a strict protocolized management and requiring more complicated algorithms. Moreover, accurate and precise monitoring of neonatal hemodynamics, using for example neonatologist performed echocardiography (NPE), non-invasive cardiac output assessment and near infrared spectroscopy (NIRS), is challenging (25). The implementation of individualized hemodynamic management in neonatal intensive care is more difficult and associated with higher variability in care (37). Nevertheless, our aim is to provide the best treatment for our patients, that takes into account the specific characteristics and cardiovascular physiology of the individual patient with the use of personal, adaptive target values for different hemodynamic variables. High quality trials are warranted to provide evidence for superiority of individualized hemodynamic management regarding short- and long-term outcomes.

## Author Contributions

This minireview is written by WB.

## Conflict of Interest

The author declares that the research was conducted in the absence of any commercial or financial relationships that could be construed as a potential conflict of interest.
